# What therapists do during guidance in individually tailored internet-based cognitive behavioral therapy for depressive symptoms: A content analysis^☆^

**DOI:** 10.1016/j.invent.2025.100875

**Published:** 2025-09-24

**Authors:** Victoria Aminoff, Laura Luisa Bielinski, Matilda Berg, Thomas Berger, Gerhard Andersson

**Affiliations:** a Department of Behavioural Sciences and Learning, Linköping University, Linköping, Sweden; b Department of Clinical Psychology and Psychotherapy, University of Bern, Switzerland; c Department of Biomedical and Clinical Sciences, Linköping University, Linköping, Sweden; d Department of Clinical Neuroscience, Karolinska Institute, Stockholm, Sweden

**Keywords:** Internet-based cognitive behavioral therapy, Individually tailored ICBT, Depression, Therapist behavior, Content analysis

## Abstract

**Introduction:**

Therapist-supported internet-based cognitive behavioral therapy (ICBT) can be effective for individuals with depressive symptoms. However, it remains unclear how therapists act when guiding individually tailored ICBT. This study examined therapist behaviors in guided ICBT where therapists choose participant modules, focusing on the proportions of behaviors and behaviors' associations with depressive symptoms and negative treatment effects.

**Methods:**

Content analysis was employed to examine 1055 messages sent from six therapists to 62 participants. *Z*-tests for proportions were used to compare therapist behaviors in the current study to those reported in studies on non-tailored ICBT. The association between therapist behavior frequencies and changes in depressive symptoms and negative effects were assessed using Spearman's rho correlation. Residual change scores were calculated for depressive symptoms.

**Results:**

All but two therapist behaviors identified in prior research on non-tailored ICBT were observed in this study. Certain behaviors such as *clarifying the framework and administrative aspects* were more common than in non-tailored ICBT for depressive symptoms. Other behaviors, such as *empathetic utterances,* were less common. The frequency of *reinforcing* correlated significantly with a decrease in depressive symptoms (*r*_s_ = −0.33, *p* = .033) and *unsupportive tone* correlated significantly with negative effects (*r*_s_ = 0.35, *p* = .022).

**Discussion:**

Almost all therapist behaviors observed in previous studies on non-tailored ICBT are also present in individually tailored ICBT, although the proportions of behaviors differ. Furthermore, the results indicate that *reinforcing* may be associated with improved treatment outcomes, while therapist *unsupportive tone* could be linked to negative effects.

## Highlights

• ICBT is effective for depressive symptoms, therapist behavior in tailored ICBT is less studied.
• Therapist behaviors in guidance messages were analyzed using content analysis.
• Most therapist behaviors found in non-tailored ICBT were seen in tailored ICBT.
• Reinforcing was associated with depressive symptoms, unsupportive tone with negative effects.
• Knowledge on therapist behavior in tailored ICBT is contributed.

## 1 Introduction

Internet-based cognitive behavioral therapy (ICBT) is an effective treatment for a wide range of mental health disorders, including depression (Andersson et al., 2019; Käll et al., 2024). When supported by the involvement of a therapist or clinician, known as guided ICBT, the approach offers many advantages. Therapist guidance has been linked to better treatment outcomes, specifically in participants with moderate to severe levels of depression (Karyotaki et al., 2021). In guided ICBT, therapists typically engage in asynchronous communication with participants, often through weekly email or chat messages. Research demonstrates that the efficacy of guided ICBT can be on par with that of traditional face-to-face therapies in controlled studies (Hedman-Lagerlöf et al., 2023) and in naturalistic settings (Rosenström et al., 2025).

Therapist-related factors are important for the effects of psychological treatments (Norcross and Lambert, 2019; Wampold and Imel, 2015). Compared to face-to-face treatments, therapist behaviors during guidance in ICBT (e.g., text messages) are always available for patients to return to and read which may potentially increase their impact, even when they are brief. Thus, the behaviors therapists exhibit during guidance interactions, may be of particular importance in ICBT (González-Robles et al., 2024). A recent scoping review (González-Robles et al., 2024) highlighted a striking imbalance: while extensive research has examined the overall effectiveness of guided ICBT, far fewer studies have explored the varied therapist behaviors that occur during guidance.

One of the earlier studies to investigate therapist guidance behaviors in ICBT for depression was conducted by Holländare et al. (2016) who identified nine therapist behavior categories and seven subcategories, ranging from *emphasise participant responsibility* to *guiding*. The authors found *affirming*, *encouraging*, and *self-disclosure* to be positively correlated with improvement in depressive symptoms at post treatment. In another study, Schneider et al. (2016) identified eleven categories of therapist behaviors in guided ICBT for depressive symptoms using both a deductive approach, based on work by Paxling et al. (2013), and an inductive approach, being open for identifying new therapist behavior categories. The identified categories ranged from *deadline flexibility* to *questions*. Several significant correlations between therapist behaviors and symptom change scores were observed. Results indicated that certain therapist behaviors were associated with worse participant outcomes (i.e. increased symptom severity from pre to post-treatment); *psychoeducation, administrative statements, self-efficacy shaping* and *task prompting*.

To date, only one study known to the authors focused on developing a scale for negative or undesirable therapist behaviors in ICBT (Hadjistavropoulos et al., 2019). The goal was to identify behaviors that have the potential to compromise ICBT treatment integrity. Six behaviors were identified: 1) *inadequate detail;* 2) *unaddressed content;* 3) *unsupportive tone*; 4) *missed correspondence*; 5) *inappropriate self-disclosure*; and 6) *unmanaged risk*. These behaviors occurred only infrequently in the analyzed emails. The most prevalent undesirable therapist behavior was *inadequate detail.* The number of undesirable therapist behaviors was not correlated with participant outcome variables.

In summary, prior research on therapist behaviors in guided ICBT for depressive symptoms show mixed findings. While some studies, such as those by Holländare et al. (2016) and Schneider et al. (2016), reported correlations between therapist behaviors and participant outcomes, Hadjistavropoulos et al. (2019) found no such associations. Moreover, little is known about the relationship between therapist behavior and symptom changes at different phases of treatment. Schneider et al. (2016) noted fewer correlations between therapist behaviors and symptom change scores from pre- to mid-treatment compared to pre- to post-treatment, while Holländare et al. (2016) examined associations between therapist behaviors in the first two weeks of treatment and depression scores. Given the distinct role of the early treatment phase in ensuring that the participant understands the treatment structure, starts to engage with the treatment content, and may start to develop a therapeutic alliance, examining therapist behaviors specifically in the early phase of treatment and comparing these behavior proportions to middle and late phases may offer valuable new insights. Similarly, examining therapist behaviors during the end of the treatment as a separate phase may be relevant as it focuses on providing the participant with an opportunity to discuss or ask questions before the therapeutic contact comes to an end or consolidating gains in preparation for the termination of treatment.

Additionally, the impact of therapist behaviors on negative treatment effects in ICBT has not been investigated. Negative effects are known to occur following most psychological treatments (Boettcher et al., 2014; Rozental et al., 2015), but this remains a relatively recent topic in ICBT research (Rozental et al., 2018). Investigating whether negative effects are linked to specific therapist guidance behaviors, particularly undesirable ones, could be valuable in preventing or mitigating these effects.

An important advancement in ICBT is the concept of treatment tailoring. Tailored ICBT often combines modules from different treatment packages, creating personalized treatment regimens based on factors such as diagnosis and comorbidity (Carlbring et al., 2011; Johansson et al., 2012). This approach has shown promise, yielding moderate-to-large effect sizes in alleviating anxiety and depression symptoms (Andersson et al., 2023; Păsărelu et al., 2017), but not necessarily a superiority over non-tailored approaches (Berger et al., 2014). One form of tailoring involves therapists selecting specific modules for participants that are deemed relevant at the start of treatment (Andersson et al., 2023). However, the role of therapist behaviors during guidance in individually tailored - through therapist-selected modules - ICBT remains unexplored. Compared to non-tailored ICBT, tailored ICBT, and particularly with therapist-selected modules, may influence the type and frequency of therapist behaviors. This is because tailored ICBT requires therapists to adjust their feedback based on individual participant progress, as participants receive different materials at varying stages of treatment. In contrast, non-tailored ICBT necessitates less adaptation. In other words, how therapists behave in the context of ICBT where they assign the treatment modules for their clients, must be examined.

The aim of this study was to investigate whether therapist behaviors identified in previous research on non-tailored ICBT for depressive symptoms are also observed in the context of individually tailored ICBT where therapists select modules for participants. Using data from the LUNA factorial trial (ClinicalTrials.gov ID, NCT04260750), a content analysis approach was employed to identify the types and proportions of therapist behaviors and compare these with the existing literature on non-tailored ICBT. The study also aimed to examine how percentages of therapist behavior vary across different phases of treatment and to explore the associations between frequencies of therapist behaviors, changes in depression symptoms from pre- to post-treatment, and the occurrence of negative treatment effects.

## 2 Method

### 2.1 Study design

Participants came from an experimental factorial design examining how support type and treatment content selection influence treatment outcomes in internet-based cognitive behavioral therapy (ICBT) for depressive symptoms (ClinicalTrials.gov ID NCT04260750). The factorial trial, named LUNA, tested three variables: type of support (scheduled support/on-demand support), selection of treatment content (self-tailored/therapist-tailored) and time (pre- and post-treatment assessment). Each component was assessed at two levels, resulting in a 2x2x2 design. The study was approved by the regional ethics review board in Linköping, Sweden. All participants provided informed consent. For the purpose of the present study, only the participants who received scheduled support and therapist-selected modules were included. This is an intentional restriction of the included participant sample, given the exploratory nature of this study, in which the aim is to initiate the investigation of therapist behaviors within individually tailored ICBT where therapists select modules.

### 2.2 Inclusion and exclusion criteria

To participate in the LUNA trial, individuals had to be over 18 years old and score at least five points on the Patient Health Questionnaire (PHQ-9; Kroenke et al., 2001) or at least ten points on the Beck Depression Inventory-II (BDI-II; Beck et al., 2005), have the ability to understand and speak Swedish and have access to the internet and a smartphone/computer. The inclusion criteria based on the depression rating scales were primarily employed as a screening tool, to identify a broader range of individuals who might potentially benefit from the treatment. Exclusion criteria included severe psychiatric disorders (e.g., psychotic disorders, substance abuse, or bipolar disorder), risk of suicidality, adjustments to psychopharmacological medications in the last three months, and ongoing psychological treatment.

### 2.3 Procedure

In the Luna trial, participants were recruited through a study website and advertising. To participate, individuals had to complete the pre-treatment measurement, consisting of demographic information, self-assessment questionnaires, questions on previous treatment experiences, and a knowledge test. Those individuals who met the inclusion criteria were then contacted by phone for the administration of the Mini International Neuropsychiatric Interview (MINI, Sheehan et al., 1998). After the interviews, the data for each participant were discussed in clinical conferences. The final decision on in- or exclusion was made by the lead project researcher (GA). More details will be reported in the controlled trial report.

### 2.4 Participants

Of the 248 individuals included in the LUNA trial, 62 were randomized to the group with scheduled support and therapist allocation of treatment content in that trial. These were the participants for which the therapist decided about their modules and guidance was provided weekly throughout the treatment, and it is these participants' therapist messages that are analyzed in the present study. Table 1 shows the demographics of these participants. Forty-two (67.7 %) of these 62 participants completed the post-treatment assessments. All messages sent by therapists to the 62 participants were examined for the analysis in this study. The therapists were six psychology students in their final term of a five-year clinical psychology program. The mean age of the therapists was 27.67 years (*SD* = 1.79) and all were female. Participants received feedback on a weekly basis and could contact their specific therapist if needed and expect an answer from the therapist within 24 h. The students who acted as therapists participated in an ICBT workshop led by the licensed psychologists within the research team. The agenda of this workshop was based on instructions compiled by the research team, drawing on prior experience from ICBT studies. In addition to guidance on how to use the treatment platform, the instructions included examples of how a message from a therapist might be formulated. However, the instructions emphasized the importance of tailoring the feedback to the individual, based on the therapist's knowledge of the participant and the participant's engagement in the treatment. The therapists also received weekly supervision from licensed psychologists during the treatment period. Communication between participants and therapists occurred through a messaging function on a secure platform (Vlaescu et al., 2016), where the treatment material was available.

**Table 1:** Participant characteristics at pre-treatment (*n* = 62).

Age, mean (*SD*; range)	29.16 (7.77; 19–63)
Female gender, *n* (%)	55 (88.7)
Education level, *n* (%)	
Compulsory school	1 (1.6)
Secondary school	14 (22.6)
Vocational school	1 (1.6)
College/university (not completed)	18 (29.0)
College/university (completed)	25 (40.3)
Other	3 (4.8)
Occupational status	
Student	17 (27.4)
Employed	34 (54.8)
Unemployed	3 (4.8)
Retired	1 (1.6)
Parental leave	3 (4.8)
Sick leave	1 (1.6)
Other	3 (4.8)
Former experience of psychological treatment, *n* (%)	48 (77.4)
Patient Health Questionnaire-9, mean (*SD*)	13.90 (5.30)

### 2.5 Intervention

The intervention lasted ten weeks and included 15 possible treatment modules based on cognitive behavioral therapy (Andersson et al., 2023). Different combinations of modules and the number of modules that was given to a participant was chosen by their therapist depending on their described problems. The content of the text-based modules comprised information and strategies targeting depression and comorbid symptoms, such as anxiety and stress. Examples are behavioral activation, cognitive restructuring, exposure, stress management, and strategies on sleep. All modules consisted of a combination of psychoeducational texts, images, and exercises. Each participant with scheduled support and therapist-selected treatment content (*n* = 62) was paired with a personal therapist. Consequently, four of the therapists were paired with ten participants each, while two of the therapists worked with eleven participants each.

Therapists' selection of modules was based on their consultation of both pre-treatment measurement and the telephone interview, including the MINI. No cut-offs or predetermined guidelines were applied to determine which modules would be included for a specific individual. Instead, the treatment plan for each participant was discussed within the research team, which included the therapists, at the time when the decision about inclusion of the participant was made. New modules were assigned weekly, except in specific cases where adjustments were deemed necessary. At the end of treatment, all included participants were asked to complete post-treatment measures and contacted for a telephone interview.

### 2.6 Support

At the start of treatment, the participants were sent two standardized messages by their therapist, one about the start of the treatment and one where the therapist introduced oneself and the treatment approach. They also received an individual treatment plan, with included modules, that was sent from the therapist. Thereafter, the routine was that therapists sent a weekly message providing feedback based on the work the participant had completed in the current module. Participants could contact their therapist at any time during the week, and the therapists responded within 24 h. Consequently, the total number of messages sent by therapists varied from week to week. Participants who did not complete any exercises received two reminders from their therapist. If they still did not complete any exercises, no further contact attempts were made, but they continued to receive emails about weekly assessments and access to modules. Thus, therapists sent messages to provide feedback on the participant's work in a module, respond to messages initiated by the participant, or remind the participant about the treatment and the therapist's availability for support. All messages from therapists to participants were included in the analysis of therapist behaviors in the present study.

### 2.7 Measures

Several self-report measures were used in the main trial. A full overview of all measures can be found on Clinicaltrials.gov (NCT04260750). In the current study, the change on the PHQ-9 (Kroenke et al., 2001) from pre-treatment to post-treatment served as the outcome measure of interest. The PHQ-9 is a measure of depressive symptoms with scores ranging from 0 to 27 (created by summing up the score from each item; Kroenke et al., 2001). Clinical cut-offs for mild, moderate, moderately severe and severe major depressive disorder are 5, 10, 15, and 20 points respectively (Kroenke and Spitzer, 2002).

The second outcome measure of interest for this study was the Negative Effects Questionnaire-20 (NEQ-20, Rozental et al., 2016). The NEQ-20 is a revised version of the NEQ-32, consisting of 20 items designed to assess negative effects of psychological treatment (Rozental et al., 2019). The questionnaire encompasses five distinct constructs: symptoms, quality, dependency, stigma, and hopelessness (Rozental et al., 2019). The items are addressed in three steps, when applicable. In the first step, the respondents are asked whether they have experienced the described phenomenon (Yes/No). If they answer “Yes”, they proceed to the second step, in which they indicate the extent to which the experience affected them on a five-point Likert scale ranging from “not at all” (0) to “extremely” (4). The total score thus ranges from 0 to 80. In the third step, respondents are asked whether the negative experience was attributable to the psychological treatment they underwent or to other life circumstances (Rozental et al., 2016).

### 2.8 Coding of therapist behaviors

A coding procedure was conducted to investigate the therapist behaviors identified in messages written by therapists supporting participants in individually tailored ICBT for depression. To this end, a directed content analysis approach as specified by Hsieh and Shannon (2005) was used. The deductive categories were derived from three previous studies on non-tailored ICBT for depressive symptoms (Hadjistavropoulos et al., 2019; Holländare et al., 2016; Schneider et al., 2016). An overview of the deductive categories used in the study can be found in Table 2, Table 3**.**

**Table 2:** Category coding system of therapist behaviors (deductive), including definitions, examples and references.

Behavior and definition	Example(s)	Adapted from	
		Holländare et al. (2016)	Schneider et al. (2016)
**Clarifying the framework and administrative aspects** Clarifying, emphasizing or reminding the participant about the treatment framework and providing administrative information and instructions to the participant.	*The treatment ends in one week (*Holländare et al., *2016**).* *I was able to share your feedback with my supervisor and our webmasters(*Schneider et al., 2016*).*	**Clarifying the framework** Clarifying, emphasizing or reminding the participant about the internet treatment framework and giving practical information about the project.	**Administrative statements** Giving the client general information about course layout, navigation, and operating procedures or announcing an upcoming check-in date with a client.
**Providing feedback and guidance** Providing the participant with advice, and also suggestions and feedback on questionnaires and submissions.	*I suggest that you start with a task that is rather simple(*Holländare et al., 2016*).* *Your scores on the depression measure indicate an improvement in symptoms since starting this course(*Schneider et al., 2016*).*	**Guiding** Giving advice, informing or making suggestions. Including subcategories theoretical guiding and giving suggestions.	**Questionnaire Feedback** Feedback given to clients about scores on website forms or other submissions and what they signify.
**Self-disclosure** Therapists mentioning personal experiences or examples relevant to the participants.	*I also get bored by physical exercise (*Holländare et al., 2016*).* *I've also had trouble sleeping (*Schneider et al., 2016*).*	**Self-disclosure** Mentioning the therapist's own experience and using personal examples from one's own life.	**Self-disclosure** Therapist behaviors that describe circumstances in the therapist's own life situation that are similar or relevant to the participant's situation.
**Psychoeducation** Psychoeducation provided by therapist including information on relevant aspects of current or upcoming material, purpose or meaning of treatment aspects.	*Worrying is part of generalized anxiety disorder (*Schneider et al., 2016*).*		**Psychoeducation** Information about psychological processes, goals of the treatment and explanation of purpose and meaning of the work involved in the treatment.
**Questions** Therapist asks for feedback or response or asks questions intended to clarify or provoke thought.	*Could you please give me a sense of how much time per day you are spending having these thoughts? (*Schneider et al., 2016*).*		**Questions** Asking clients a question for the purposes of clarifying a statement made by the therapist or asking the client a question intended to provoke thought about their life situation as it relates to course material.
**Reinforcing** Behaviors aimed at reinforcing what a participant has done concerning specific tasks (including praise for completed tasks).	*Good of you to notice your own feelings in that situation!* *(*Holländare et al., 2016*)* *You've described your worry* *thoughts in a good way (*Schneider et al., 2016*)*	**Encouraging** Therapist behaviors aimed at encouraging some type of participant behavior including praising past behavior and inciting future behavior.	**Task reinforcemen**t Behaviors aimed at reinforcing assignments already completed by the participant.
**Prompting** Telling the participants to do something, urging and prompting them.	*Please fill out the weekly rating (*Holländare et al., 2016*).* *I'm looking forward to hearing from you during the work with the coming modules (*Schneider et al., 2016*).*	**Urging** Urging the participant to do something.	**Task prompting** Behaviors prompting the participant to work with a given homework assignment and explicit interest in future results of the participant's progress.
**Empathetic utterance** Therapist conveys that they know or understand how the participant feels.	*We all have our ups and downs (*Holländare et al., 2016*)* *I can see why you (*Schneider et al., 2016*).*	**Affirming** Paying attention to, acknowledging and expressing an interest in, the participants' thoughts, emotions and actions and to deem them valid. Including subcategories: validating and interpreting, normalizing and summarizing.	**Empathetic utterance** Writings that attempt to convey understanding and empathy for the participant's suffering, frustration, or general life situation.
**Alliance bolstering** Non-treatment specific writings that pertain to interest in the participant's life situation and care for their situation.	*How nice that you've had a good week (*Schneider et al., 2016*).*		**Alliance bolstering** Non-treatment specific writings that pertain to interest in the participant's life situation and care for his or her situation.
**Confronting** Expressing another opinion or disagreeing with the participant.	*As a first exercise, that seems too difficult (*Holländare et al., 2016*).*	**Confronting** Expressing another opinion or disagreeing with the participant.	
**Deadline flexibility** Behaviors that pertain to lenience from the therapist concerning deadlines for homework submissions and allowance of extra time to work with a given module.	*You'll get another couple of days to finish the task (*Schneider et al., 2016*).*		**Deadline flexibility** Behaviors that pertain to lenience from the therapist concerning deadlines for homework submissions and allowance of extra time to work with a given module.
**Emphasizing participant responsibility** Expressing that the participant is responsible for (among other things) their own decisions.	*You yourself have to determine what is best(*Holländare et al., 2016*).*	**Emphasizing participant responsibility** Expressing that the participant is responsible for (among other things) his/her own decisions.	
**Self-efficacy shaping** Behaviors that prompt and reinforce the participant to spontaneously engage in the health prompting behaviors they have learnt through the treatment.	*The more you practice this, the more often you'll be able to notice the thoughts (*Paxling et al., 2013*;*Schneider et al., 2016*).*		**Self-efficacy shaping** Behaviors that prompt and reinforce the participant to spontaneously engage in the health prompting behaviors they have learnt through the treatment.
**Informing about modules** Informing about, or making reference to, upcoming, or earlier, modules and module content.	*Module 6 will cover…(*Holländare et al., 2016*).*	**Informing about modules** Informing about, or making reference to, upcoming, or earlier, modules and module content.	

*Note*. The table shows our category system alongside the original categories present in the works of Holländare et al., 2016 and Schneider et al., 2016. The word *patient* was exchanged with the word *participant*. 8 of 11 categories used by Schneider were originally proposed by Paxling et al. (2013).

**Table 3:** Category coding system of undesirable therapist behaviors (deductive), including definitions, examples and references.

Behavior and definition	Detailed description
**Inadequate detail** Message is extremely short and is missing a large proportion of the qualities that are expected in a therapist message.	Therapist writes a brief message (i.e. one short paragraph) that includes no symptom feedback, or indication when the next lesson will become available.
**Unaddressed content** Therapist ignores or does not address comment or question from previous participant message.	Therapist fails to answer a question about cognitive restructuring asked in the participant message. Therapist makes no reference to any content from the participant's most recent message.
**Unsupportive tone** Tone of message is unsupportive or critical.	Therapist bluntly states that participant has logged in for many weeks without providing a statement of support (e.g., *“I see that you have not logged in on 17 days”*).
**Missed correspondence** Therapist misses a scheduled communication without providing advanced warning.	Therapist is unable to send their scheduled message due for example, a sudden illness but no message is sent to the participant informing them of the therapist's absence.
**Inappropriate self-disclosure** Therapist self-disclosure that has no clear therapeutic purpose or detracts from the participant's situation.	Therapist makes reference to their own anxiety or depressive symptoms *without connecting it in a clear way to the participant's situation or making it part of a clear way of normalizing.*
**Unmanaged risk** Therapist does not appropriately address an increase in symptom severity or suicidality.	Therapist mentions an increase in suicidal ideation in their message, but does not phone participant to follow up.

*Note*. All behaviors and descriptions are derived from the publication by Hadjistavropoulos et al. (2019). The term Email was replaced by message. For inappropriate self-disclosure the description was expanded to include: *without connecting it in a clear way to the participant's situation or making it part of a clear way of normalizing.*

Alongside the deductive approach, the authors were open to the possibility of finding novel therapist behaviors specific to tailored ICBT by forming inductive categories.

To prepare the data for analysis, messages from the therapists (*n* = 6) were exported from the treatment platform and anonymized. Following the methodology employed by Schneider et al. (2016) and Holländare et al. (2016), portions of, entire, and multiple sentences within the messages were analyzed as units to capture the function of the behavior portrayed in the text. Thus, consecutive behaviors addressing the same topic were treated as interconnected and categorized under a single therapist behavior. In addition, conversely, if a behavior (e.g. one sentence) addressed multiple distinct purposes, it could be assigned multiple categories.

Two authors (LLB and VA) developed the coding guide based on prior categories described by Schneider et al. (2016), Holländare et al. (2016), and Hadjistavropoulos et al. (2019). All categories were thoroughly discussed, and many of the definitions previously formulated were utilized. However, adjustments were made to the definitions when necessary to fit the current data, conducted in collaboration with all authors (see Table 2). Categories assessed as overlapping between Schneider et al. (2016) and Holländare et al. (2016) were consolidated into a single category with a combined definition. One author (VA) started by coding ten randomly selected participants' messages from their therapists to investigate the usefulness of the developed coding guide. In cases of ambiguity, codes were discussed and determined through consensus among all authors. Subsequently, two authors (VA and MB) independently coded the same therapist messages (*n* = 186) from the ten participants to assess interrater-reliability. Cohen's Kappa for the cross-coded messages was K = 0.96, which is interpreted as almost perfect agreement (McHugh, 2012) and comparable to other studies in the field (Paxling et al., 2013; Schneider et al., 2016). Thus, the interrater reliability was at a level that ensures that the coding guide can be used and applied similarly by different coders. One author (VA) coded the remaining messages. Any ambiguities or the potential identification of new codes were discussed with all authors.

As the coding scheme was developed by the research team, based on previous research (Hadjistavropoulos et al., 2019; Holländare et al., 2016; Schneider et al., 2016), and the data was analyzed by the same team, a reflection on researcher reflexivity is warranted. The research team comprises members of varying ages, genders, and levels of research experience. GA and TB are professors of psychology, MB and LLB hold PhDs, and VA is a PhD student. All members have experience with online intervention treatment and research. GA, TB, and MB have extensive experience with qualitative methodology within the ICBT research field. All members reviewed and familiarized themselves with the studies forming the basis of the coding guide used in this study, and approached the data and codes in light of these earlier works as well as prior knowledge from other studies in the field.

### 2.9 Quantitative analysis

Quantitative analyses were conducted with IBM SPSS Statistics 29 (IBM, New York) and R (Version 4.4.2; R Core Team, 2024). To compare therapist behaviors between our study and previous work (Hadjistavropoulos et al., 2019; Holländare et al., 2016; Schneider et al., 2016), two-tailed z-tests for proportions were conducted. If frequencies of behavior were very low, Fisher's exact test was also conducted. Beginning, middle and end of treatment were defined as the first two weeks, weeks three to eight, and weeks nine to ten of treatment, respectively. For the intercorrelations between therapist behaviors, Spearman's rho, two-tailed, was calculated. Spearman's rho was interpreted as a small (*r*_s_ > 0.10), moderate (*r*_s_ > 0.30), or large (*r*_s_ > 0.50). Missing data for the PHQ-9 and NEQ-20 at post-treatment were not imputed and a Complete Case Analysis approach was employed (*n* = 42). Correlations between therapist behavior frequencies and PHQ-9 change scores were calculated using Spearman's rho and using residual change scores for the PHQ-9. These were calculated with the formula Z_2_ − (Z_1_ ∗ R_12_). Correlations between frequency of therapist behaviors and NEQ-20 total score were also calculated using Spearman's rho. For all analyses, alpha level at 0.05 or 0.01 was used. Differences in percentages of therapist behaviors between different phases of treatment and between the completer and dropout samples were reported descriptively. Completers were defined as those participants that completed the post-treatment measurement, whereas non-completers did not complete the post-treatment measurement.

## 3 Results

On average, participants logged in to the treatment platform 19.71 times (*SD* = 14.39) and sent an average of 3.60 messages (*SD* = 3.52) to their therapists. Therapists, in turn, sent an average of 17.02 messages (*SD* = 4.81) to the participants. In total, 1055 messages were sent from the therapists to the participants during the treatment **(**Table 4**).**

**Table 4:** Number and percentage of therapist behaviors found in the 1055 messages.

Therapist behavior	*n*	%
Clarifying the framework and administrative aspects	1499	25.59
Providing feedback and guidance	795	13.57
Self-disclosure	17	0.29
Psychoeducation	199	3.40
Questions	271	4.63
Reinforcing	567	9.68
Prompting	1202	20.52
Empathetic utterance	242	4.13
Alliance bolstering	813	13.88
Confronting	7	0.12
Deadline flexibility	50	0.85
Emphasizing participant responsibility	10	0.17
Self-efficacy shaping	74	1.26
Informing about modules	84	1.43
Inadequate detail	2	0.03
Unaddressed content	1	0.02
Unsupportive tone	2	0.03
Missed correspondence	23	0.39
Inappropriate self-disclosure	0	0
Unmanaged risk	0	0
Total	5858	100

Within these messages, 5858 therapist behaviors were identified that belonged to 18 distinct categories. The most frequent therapist behavior was *clarifying the framework and administrative aspects* (25.59 % of all behaviors), followed by *prompting* (20.52 %), *alliance bolstering* (13.88 %), and *providing feedback and guidance* (13.57 %). Many of the frequencies of the therapist behaviors were highly correlated with each other (see Supplementary data, Appendix A). However, none or very few significant correlations were found for the behaviors *self-disclosure*, *confronting*, *deadline flexibility* and the undesirable behaviors.

All therapist behaviors reported by Schneider et al. (2016) and Holländare et al. (2016) were also observed in this study (see Supplementary data, Appendix B and C). However, of the six undesirable therapist behaviors identified by Hadjistavropoulos et al. (2019), only four were detected and these were relatively infrequent (see Supplementary data, Appendix D). Significant differences in proportions of therapist behaviors between our study and the non-tailored ICBT studies were found for all but four behaviors (Supplementary data, Appendix B, C, and D).

### 3.1 Therapist behaviors in different phases (beginning, middle, end) of treatment

The counts and percentages of therapist behaviors at the beginning, middle and end of treatment are displayed in full in the Supplementary data, Appendix E. The most common behavior at the beginning, middle and end of treatment was *clarifying the framework and administrative aspects*, which corresponded to 33 %, 21 %, and 25 %, respectively. Thus, the most common behavior was the same across all treatment phases, whereas *prompting* was the second most common behavior in the beginning (26 %) and middle (18 %), while *alliance bolstering* ranked second in the end of treatment (24 %), while *prompting* also remained relatively common in the end (18 %).

### 3.2 Therapist behaviors for completer and dropout samples

When comparing therapist behaviors between completer and dropout samples, for example, lower percentages of c*larifying the framework and administrative aspects* and *prompting* occurred in the completer sample than in the dropout sample. In contrast, for examp higher percentages of *providing feedback and guidance, reinforcing*, and *empathic utterances* occurred in the completer sample. The full table of comparison can be found in the Supplementary data, Appendix F. These differences are reported descriptively only; no inferential statistical tests were conducted due to non-independence of therapist data between groups.

### 3.3 Correlations between therapist behavior and outcomes

When examining the correlation between the frequency of therapist behaviors and the change in self-reported depressive symptoms from pre- to post-treatment, a moderate correlation was identified for *reinforcing* (*r*_s_ = −0.33, *p* = .033). This indicates that *reinforcing* behaviors were associated with reductions in self-reported depressive symptoms. No other therapist behavior was found to significantly correlate with changes in self-reported depressive symptoms during the whole course of treatment.

When investigating the correlation between the frequency of the therapist behaviors early in the treatment and changes in depressive symptoms, three therapist behaviors were found to be significantly correlated with symptom change: *providing feedback and guidance* (*r*_s_ = −0.31, *p* = .009), *reinforcing* (*r*_s_ = −0.33, *p* = .033), and *empathetic utterance* (*r*_s_ = −0.34, *p* = .029). All correlations were moderate in strength. None of the frequencies of therapist behaviors during the middle or end of the treatment were significantly correlated with changes in depressive symptoms.

Regarding the correlation between the frequency of therapist behaviors and negative effects, it was found that the therapist behavior *unsupportive tone* was moderately correlated with reported negative effects (*r*_s_ = 0.35, *p* = .022). This indicates that a higher frequency of *unsupportive tone* in total during the treatment was associated with higher NEQ-20 scores. No other significant correlations were observed between therapist behavior and NEQ-20, either for therapist behaviors in total throughout the entire treatment or specifically during different phases of the treatment.

## 4 Discussion

The aim of this study was to explore therapist behaviors in tailored ICBT for depression where therapists selected treatment modules. All therapist behaviors identified by Schneider et al. (2016) and Holländare et al. (2016) were observed. However, of the undesired therapist behaviors outlined by Hadjistavropoulos et al. (2019), *inappropriate self-disclosure* and *unmanaged risk* were notably absent. Despite also employing an inductive approach to enable the identification of therapist behaviors that might not fit the behaviors previously described by Schneider et al. (2016), Holländare et al. (2016), or Hadjistavropoulos et al. (2019), no new behaviors were identified. These findings indicate that the type of behavior employed in therapist guidance messages for individually tailored ICBT may not differ from the type of behaviors in guidance messages for non-tailored ICBT.

At the same time, the proportions of all but four behaviors differed significantly between this study and previous work (Hadjistavropoulos et al., 2019; Holländare et al., 2016; Schneider et al., 2016). For example, the proportion of *clarifying the framework and addressing administrative aspects* was higher in our study than in the studies by Holländare et al. (2016) and Schneider et al. (2016). Perhaps, tailored ICBT requires more clarification of the framework than non-tailored ICBT due to therapists choosing specific modules for participants. As a second example, the proportions of *inadequate detail*, *unaddressed content,* and *inappropriate self-disclosure* were significantly lower in our study than in the work presented by Hadjistavropoulos et al. (2019), while there was more *missed correspondence* in our study. This is exploratory work, and future studies would need to replicate the findings on undesired therapist behaviors to validate potential differences between tailored and non-tailored ICBT.

Regarding the correlations between frequency of therapist behaviors and depressive symptom change scores, an overall higher frequency of *reinforcing* was associated with larger reductions in self-reported depressive symptoms in our study. As with all correlations, the observed association between the total frequency of *reinforcing* throughout treatment and greater reductions in depressive symptoms can be interpreted from different perspectives while causality cannot be inferred. On one hand, *reinforcing* may have direct therapeutic effects or enhance participant engagement, thereby increasing adherence to the treatment which has previously been shown effective for depressive symptoms (Andersson et al., 2023). On the other hand, symptom reduction itself may influence participant message behavior or participant engagement with treatment modules, prompting therapists to use *reinforcing* more frequently. These results align with findings from Holländare et al. (2016), who identified a similar correlation between the therapist behavior *encouraging* and reductions in depressive symptoms pre- to post-treatment. However, the observed correlation between *reinforcing* and depressive symptom reduction contrasts with Schneider et al. (2016), who found no significant association between *task reinforcement* and depressive symptom reduction.

Consistent with Holländare et al. (2016) who examined therapist behaviors during the first two weeks of treatment, our findings indicate an early-treatment correlation between *reinforcing* and reductions in depressive symptoms. Additionally, the frequency of *providing feedback and guidance* and *empathetic utterance* in the beginning of the treatment correlated with depressive symptom reduction in our study, unlike in the studies of Holländare et al. (2016) and Schneider et al. (2016). This may suggest that these behaviors play a more critical role at the start of tailored ICBT with therapist-chosen modules, where treatment structure may be perceived as less explicit compared to non-tailored ICBT. Consequently, participants receiving more feedback and guidance might experience greater symptom reduction. Alternatively, symptom reduction itself could elicit increased therapist feedback and guidance, particularly in early treatment, when participants may benefit more from such support. As treatment progresses, this need may diminish, potentially explaining why no therapist behaviors during mid- or end of the treatment correlated with symptom reduction. In the trial, therapists selected and granted access to the most relevant treatment modules at the beginning of the treatment. Consequently, therapist behaviors associated with these initial modules may have offered greater support than those in later treatment stages.

Examining therapist behaviors at different phases of treatment, perhaps particularly at the beginning, is relevant as the early sessions appear important for setting the course of treatment and therapeutic alliance (del Río Olvera et al., 2022). In this study, the therapist behaviors *providing feedback and guidance*, *reinforcing*, and *empathic utterance* were found to be correlated with symptom reduction during the beginning of treatment, highlighting their potential relevance. As therapeutic alliance has been shown in some studies to be associated with better treatment outcomes (Pihlaja et al., 2018) and as a mediator for the relationship between guidance and outcome in internet-based treatment (Bur et al., 2022), a next step could be to investigate the relation between these early therapist behaviors in ICBT, symptom reduction, and the development of therapeutic alliance.

In contrast to Schneider et al. (2016), we found no therapist behaviors correlated with depressive symptom increase. This suggests that the therapist behaviors examined in our study may not be associated with symptom deterioration or that depressive symptoms deterioration do not seem to trigger specific therapist behaviors. Interestingly*, unsupportive tone* was moderately correlated with reported negative effects scores (*r*_s_ = 0.35, *p* = .022). This indicates that a higher frequency of *unsupportive tone* is associated with higher NEQ-20 scores and vice versa. When developing the NEQ-20, Rozental et al. (2016) found lack of quality in the therapeutic relationship as one of the factors associated with the highest self-reported negative effect. Text-based online interactions can create a therapeutic alliance between therapist and participant (Cook and Doyle, 2002), but an unsupportive tone by the therapist may be particularly impactful for individuals with depression, as negative interpretation biases are a core feature of the disorder (Cowden Hindash and Amir, 2012). This elucidates the potential impact of therapist tone on perceived negative effects, potentially underscoring the importance of maintaining a supportive and empathetic communication style in ICBT. There are several techniques suggested to improve the emotional impact of a message, but they are not well researched (Paul et al., 2017). Even if emoticons and smileys have been reported to influence the emotional impact of a message, they can only convey a limited number of emotions, and other techniques have not been investigated. Thus, techniques for conveying emotions in text-based internet communication need to be examined further in health care settings (Paul et al., 2017). In contrast, other undesirable therapist behaviors, such as *inadequate detail*, did not correlate with negative treatment effects in our study. Possibly it is because participants could compensate by reviewing the treatment material or seeking clarification by asking. However, it must be noted that the limited frequency of identified negative therapist behaviors may have influenced the lack of further correlations with negative effects. Additionally, no other therapist behaviors correlated with negative effects in our study, suggesting that these may not be associated with adverse treatment outcomes.

Finally, descriptive data shows a lower percentage of *clarifying the framework and administrative aspects* and *prompting* in the completer sample than in the dropout sample. This could mean that therapists try to clarify and prompt more if they sense that participants are dropping out. Or, though perhaps less likely, that participants drop out more when therapists clarify or prompt more. Moreover, percentages of therapist behaviors *self-disclosure, questions, alliance bolstering, confronting, inadequate detail* and *missed correspondence* were higher in the dropout sample than in the completer sample. These findings remain solely descriptive in nature and thus warrant further investigation.

### 4.1 Strengths and limitations

This study contributes to the research field of therapist behaviors in guided ICBT. Notably, it investigates therapist behaviors within a tailored version of ICBT, where therapists have the autonomy to select the treatment content. It also examines the frequency of therapist behaviors across different phases of treatment and the association with changes in depressive symptoms. Importantly, our study adds to the literature by examining therapist guidance behaviors in connection with participant reported negative effects.

A first limitation of our work concerns the content analysis. We applied categories derived from three seminal studies on therapist behaviors in ICBT and took the liberty of combining overlapping categories. Other research groups might have chosen different deductive frameworks or grouped the categories differently. We limited our analysis to behavior categories from studies specifically examining ICBT for depressive symptoms, along with the Undesirable Therapist Behaviors Scale (Hadjistavropoulos et al., 2019) where not every participant in the study had depressive symptoms. Moreover, studies on therapist behavior in ICBT for other disorders, disorder combinations, or client populations have also identified other potential therapist behaviors such as personal address, forging therapy integrity or promoting empowerment/instilling hope (Berg et al., 2022; de Bruin and Meijer, 2017; O'Brien, 2018). Additionally, the coding results may have varied with a different level of granularity of the coding process. We analyzed, in line with Holländare et al. (2016) and Schneider et al. (2016), therapist texts based on their meaning units or intended purposes, often assigning codes to entire sentences or segments of sentences, though some codes were applied to individual words. The level of coding—whether at the word, sentence, paragraph, or full-message level—would likely yield different insights. Moreover, both deductive and inductive approaches were employed in the study. The code guide, and thus the deductive framework applied, appears to be robust. While this may suggest that therapist behaviors in tailored ICBT are similar to those in non-tailored ICBT, it may also indicate that the code guide restricted the inductive approach. Consequently, it is plausible that new codes, falling outside the scope of the code guide, might have been identified if the data had been analyzed inductively to a greater extent. This highlights a potential next step for future research in the field, in which therapist behaviors could be analyzed inductively and subsequently compared with previously defined codes from deductive systems.

A second limitation concerns, that individually tailored ICBT can take many forms, not solely based on therapist-chosen treatment modules. Other customization methods might include client-tailored modules (Andersson et al., 2023), a combination of therapist- and client-tailored modules (Aminoff et al., 2023; Lindhe et al., 2023) or other variations, and as such, the findings from this study may not be applicable to all types of tailored ICBT. The same applies to the type of therapist support. In this study, we focused on therapist messages sent in a weekly support condition. However, the LUNA trial also included participants randomized to support on demand, in which participants did not have weekly support but could contact their therapist when needed. This limits the generalizability of the present findings to other forms of therapist support. To our knowledge, this is the first study to examine therapist behaviors within individually tailored ICBT, and more comprehensive comparisons would have been required if multiple groups receiving different types of support had been included. Nonetheless, this represents a valuable future direction for research in this field, particularly for advancing the understanding of the therapist's role in ICBT. Thus, future research could favorably compare therapist guidance behaviors across different versions of tailored ICBT.

A third limitation concerns the relatively small sample size. Even if the number of therapist messages analyzed in the present study (*n* = 1055) are comparable with other studies (e.g. Schneider et al., 2016), a larger sample of therapist-participant interactions could potentially reveal additional therapist behaviors. The low frequency of certain behaviors, for example, *unsupportive tone*, in our data makes it challenging to draw conclusions about their association with changes in depressive symptoms or the occurrence of negative effects. Furthermore, as is characteristic of correlational analyses, we cannot make causal inferences nor account for the potential influence of third variables on the observed associations. Regarding the descriptive comparisons of completer and dropout samples, future analyses may also want to use statistical models that account for nested structure of the data, such as mixed-effects logistic regression or generalized estimating equations (GEE) to analyze the differences between completers and dropouts in more detail.

Lastly, while this study employed a deductive approach with the aim of comparing therapist behaviors to those reported in non-tailored ICBT, caution is needed when making such comparisons. Variations in results, such as discrepancies in the correlation between therapist behavior frequencies and depressive symptom changes, may be attributable to differences in the depression questionnaires used. For example, while Holländare et al. (2016) utilized the Montgomery-Åsberg Depression Rating Scale-Self Rated (MADRS-S, Svanborg and Åsberg, 1994), we used the PHQ-9. Differences in proportions of behaviors could also be due to the method of combining therapist behavior categories that were similar from Holländare et al. (2016) and Schneider et al. (2016), potentially resulting in a larger proportion of certain defined behaviors in our study than in the comparisons.

### 4.2 Future directions

Several aspects pertaining to the findings presented, would be valuable to explore in future research. For instance, developments in artificial intelligence (AI) are advancing rapidly within the field of internet-based psychological interventions (Beg et al., 2024; Beg, 2025; Löchner et al., 2025) and may be useful tools to make a content analysis approach more efficient and precise. For example, the behavioral codes and frequencies we documented in this analysis could serve as a vital blueprint for training and refining the next generation of therapeutic AI, helping to ensure that automated systems are grounded in empirically-observed human therapeutic interactions.

To investigate and establish causal relationships, including those between therapist behaviors and treatment outcomes, randomized designs would be required. Such designs would allow for comparisons between different types of therapist support or guidelines for providing support. Given that no new therapist behaviors were identified in our data beyond those previously defined by Holländare et al. (2016), Schneider et al. (2016) and Hadjistavropoulos et al. (2019), it could be relevant to further examine therapist behaviors in clinical settings and assess whether they differ from those identified in more controlled research studies. This is particularly relevant for evaluating the ecological validity of existing findings. Lastly, this is, to our knowledge, the first study to examine the relationship between undesirable therapist behaviors in ICBT (Hadjistavropoulos et al., 2019) and negative effects (Rozental et al., 2016). Further investigation would be valuable to replicate findings on specific therapist behaviors that may be related to different types of negative effects in ICBT.

### 4.3 Conclusion

Many therapist behaviors identified in non-tailored ICBT can also be identified in tailored ICBT. However, the proportions of most behaviors differed between the two treatment types. The therapist behavior *reinforcing* showed to be associated with decreased depressive symptoms, while *unsupportive tone* was related to negative effects.Further research is necessary to substantiate these findings. Pursuing this line of research further, could be especially relevant for the development of guidelines for therapist behavior during ICBT.

## Funding statement

This research was funded in part by the Swedish Science foundation and a professors grant to Professor Andersson.

## Declaration of competing interest

We have nothing to declare.

## Data Availability

The data presented in this study is derived from messages to participants containing sensitive data and, therefore the messages, cannot be made publicly available. For examples of therapist behaviors within each category, please contact the corresponding author. Also, the frequency of therapist behavior raw data will be provided upon reasonable request.

## Supplementary data

Supplementary material Image 1
