# Supplementary material for: What therapists do during guidance in individually tailored internet-based cognitive behavioral therapy for depressive symptoms: A content analysis
Source: Internet Interv. 2025 Sep 24;42:100875. doi: 10.1016/j.invent.2025.100875 (PMC12495253; doi:10.1016/j.invent.2025.100875)
Supplement: Supplementary file 1 — Supplementary material [file mmc1.docx]

**Supplementary material**

**Appendix A**

Intercorrelations for therapist behaviors using Spearman’s rho.

| Behavior code | 1 | 2 | 3 | 4 | 5 | 6 | 7 | 8 | 9 | 10 | 11 | 12 | 13 | 14 | 15 | 16 | 17 | 18 | *M* | *SD* |
| --- | --- | --- | --- | --- | --- | --- | --- | --- | --- | --- | --- | --- | --- | --- | --- | --- | --- | --- | --- | --- |
| 1.Clarifying the framework and administrative aspects | - |  |  |  |  |  |  |  |  |  |  |  |  |  |  |  |  |  | 24.18 | 5.48 |
| 2.Providing feedback and guidance | .61*** | - |  |  |  |  |  |  |  |  |  |  |  |  |  |  |  |  | 12.82 | 10.01 |
| 3.Self-disclosure | .13 | .00 | - |  |  |  |  |  |  |  |  |  |  |  |  |  |  |  | 0.27 | 0.61 |
| 4.Psychoeducation | .53*** | .65*** | .29* | - |  |  |  |  |  |  |  |  |  |  |  |  |  |  | 3.21 | 3.12 |
| 5.Questions | .36** | .63*** | -.04 | .48*** | - |  |  |  |  |  |  |  |  |  |  |  |  |  | 4.37 | 2.73 |
| 6.Reinforcing | .64*** | .85*** | .12 | .71*** | .61*** | - |  |  |  |  |  |  |  |  |  |  |  |  | 9.15 | 7.86 |
| 7. Prompting | .66*** | .42*** | .19 | .46*** | .15 | .54*** | - |  |  |  |  |  |  |  |  |  |  |  | 19.39 | 5.35 |
| 8.Empathetic utterance | .39** | .74*** | .12 | .66*** | .45*** | .68*** | .30* | - |  |  |  |  |  |  |  |  |  |  | 3.90 | 3.55 |
| 9.Alliance bolstering | .54*** | .69*** | -.10 | .58*** | .44*** | .57*** | .31* | .62*** | - |  |  |  |  |  |  |  |  |  | 13.11 | 7.35 |
| 10.Confronting | .14 | -.05 | .05 | .10 | .06 | .03 | -.08 | -.06 | -.09 | - |  |  |  |  |  |  |  |  | 0.11 | 0.32 |
| 11.Deadline flexibility | .25* | .16 | -.22 | .10 | .10 | .06 | .12 | .11 | .17 | -.01 | - |  |  |  |  |  |  |  | 0.81 | 1.30 |
| 12.Emphasizing participant responsibility | .24 | .30* | .07 | .32* | .25* | .20 | .25* | .26* | .14 | .03 | -.06 | - |  |  |  |  |  |  | 0.16 | 0.49 |
| 13.Self-efficacy shaping | .40** | .74*** | -.12 | .54*** | .37** | .71*** | .38** | .55*** | .39** | .01 | .19 | .09 | - |  |  |  |  |  | 1.19 | 1.54 |
| 14.Informing about modules | .32* | .52*** | .33** | .59*** | .25 | .45*** | .29* | .39** | .37** | -.03 | .02 | .30* | .30* | - |  |  |  |  | 1.35 | 1.49 |
| 15.Inadequate detail | .17 | .12 | -.09 | .22 | -.02 | .10 | .02 | .15 | .13 | .22 | .07 | .21 | .16 | .13 | - |  |  |  | 0.03 | 0.18 |
| 16.Unaddressed content | -.05 | .16 | -.07 | .09 | .18 | .05 | -.15 | .22 | .21 | -.05 | -.10 | -.05 | .11 | .16 | -.02 | - |  |  | 0.02 | 0.13 |
| 17.Unsupportive tone | .27* | .11 | .12 | .07 | -.02 | .10 | .24 | -.01 | .05 | .22 | .00 | -.07 | .07 | .02 | -.03 | -.02 | - |  | 0.03 | 0.18 |
| 18.Missed correspondence | .11 | .11 | .27* | .15 | .34** | .05 | .16 | .27* | .13 | -.02 | -.14 | .26* | -.20 | .27* | -.12 | .26* | .07 | - | 0.37 | 0.66 |

Note: * p < .05, ** p < .01, *** p < .001. The codes “Inappropriate self-disclosure” and “Unmanaged risk” were not once identified in the data and, consequently, are not included in the table. Therapist behavior mean and SD for each participant are also shown.

**Appendix B**

Comparison of behavior frequencies of the current study to those reported by Schneider et al. 2016.

| ***Current study***  *n = 5729* | | | ***Schneider et al. (2016)***  *n = 9085* | | |  |
| --- | --- | --- | --- | --- | --- | --- |
| Behavior category | *n* | *%* | Behavior category | *n* | *%* | *z-score* |
| *Clarifying the framework and administrative aspects* | *1499* | 26.2 | *Administrative statements* | - | *16* | **15.13** |
| *Providing feedback and guidance* | *795* | 13.9 | *Questionnaire Feedback* | - | *3* | **24.97** |
| *Self-disclosure* | *17* | 0.3 | *Self-disclosure* | *55* | *1* | **-2.63** |
| *Psychoeducation* | *199* | 3.5 | *Psychoeducation* | *949* | *10* | **-15.46** |
| *Questions* | *271* | 4.7 | *Questions* | - | *9* | **-9.78** |
| *Reinforcing* | *567* | 9.9 | *Task reinforcement* | *1275* | *14* | **-7.43** |
| *Prompting* | *1202* | 21.0 | *Task prompting* | *1265* | *14* | **11.23** |
| *Empathetic utterance* | *242* | 4.2 | *Empathetic utterance* | *718* | *8* | **-8.86** |
| *Alliance bolstering* | *813* | 14.2 | *Alliance bolstering* | *1941* | *21* | **-10.93** |
| *Deadline flexibility* | *50* | 0.9 | *Deadline flexibility* | *61* | *1* | 1.38 |
| *Self-efficacy shaping* | *74* | 1.3 | *Self-efficacy shaping* | *219* | *2* | **-4.76** |

*Note.* Bold indicates significance at p < .01. Counts and corresponding percentages were taken directly from Schneider et al. (2016). When exact counts were not reported, z tests were calculated using the published percentage values converted to proportions. Percentages from the present study were rounded to one decimal place.

**Appendix C**

Comparison of behavior frequencies in the current study to those reported by Holländare et al. 2016.

| ***Current study***  *N total = 4423* | | | ***Holländare et al. (2016****)*  *N total = 3530* | |  |
| --- | --- | --- | --- | --- | --- |
| Behavior category | *n* | *%* | Behavior category | *%* | *z-score* |
| *Clarifying the framework and administrative aspects* | *1499* | 33.9 | Clarifying the framework | *5.9* | **30.21** |
| *Providing feedback and guidance* | *795* | 18.0 | *Guiding* | *22.2* | **-4.66** |
| *Self-disclosure* | *17* | 0.4 | *Self-disclosure* | *0.9* | **-2.81** |
| *Reinforcing* | *567* | 12.8 | Encouraging | *31.5* | **-20.31** |
| *Prompting* | *1202* | 27.2 | *Urging* | *9.8* | **19.47** |
| *Empathetic utterance* | *242* | 5.5 | *Affirming* | *25.1* | **-24.88** |
| *Confronting* | *7* | 0.2 | *Confronting* | *0.4* | -1.65 |
| *Emphasizing participant  responsibility* | *10* | 0.2 | *Emphasizing participant  responsibility* | *0.7* | **-3.42** |
| *Informing about modules* | 84 | 1.9 | *Informing about modules* | *3.4* | **-4.20** |

*Note.* Bold indicates significance at p < .01. Percentages were taken directly from Holländare et al. (2016); z tests were calculated using the reported percentage values converted to proportions. Percentages from the present study were rounded to one decimal place.

**Appendix D**Comparison of behavior frequencies in the current study to those reported by *Hadjistavropoulos et al. 2019.*

|  | ***Current study*** *N total =1053 emails* | | ***Hadjistavropoulos et al. (2019)***  *N total = 720 emails* | |  |
| --- | --- | --- | --- | --- | --- |
|  | *n* | *%* | *n* | *%* | *z-score* |
| *Inadequate detail* | *2* | 0.2 | *46* | *6.4* | **-7.90** |
| *Unaddressed content* | *1* | 0.1 | *29* | *4.0* | **-6.31** |
| *Unsupportive tone* | *2* | 0.2 | *4* | *0.6* | -1.30 |
| *Missed correspondence* | *23* | 2.2 | *4* | *0.6* | **2.75** |
| *Inappropriate self-disclosure* | *0* | 0 | *4* | *0.6* | -2.42* |
| *Unmanaged risk* | *0* | 0 | *2* | *0.3* | -1.71 |

*Note*. For comparison with Hadjistavropoulos et al. (2019), percentages were calculated as proportions of all emails (total n), rather than as proportions of all therapist behaviors. For z tests, p < .05 is indicated with an asterisk, and p < .01 is indicated in bold. Because of low cell counts, Fisher’s exact tests (two-tailed) were also conducted; the significance results did not differ from those obtained with the z tests.

**Appendix E**

Counts and percentages of therapist behaviors by treatment phase

| ***Therapist behavior*** | ***Beginning of treatment*** *(n = 1972)* | | ***Mid-treatment*** *(n= 3081 )* | | ***End of treatment*** *(n= 805)* | |
| --- | --- | --- | --- | --- | --- | --- |
|  | *n* | *%* | *n* | *%* | *n* | *%* |
| *Clarifying the framework and administrative aspects* | 649 | 32.91 | 647 | 21 | 203 | 25.22 |
| *Providing feedback and guidance* | 186 | 9.43 | 503 | 16.33 | 106 | 13.17 |
| *Self-disclosure* | 8 | 0.41 | 9 | 0.29 | 0 | 0 |
| *Psychoeducation* | 44 | 2.23 | 143 | 4.64 | 12 | 1.49 |
| *Questions* | 64 | 3.25 | 187 | 6.07 | 20 | 2.48 |
| *Reinforcing* | 135 | 6.85 | 367 | 11.91 | 65 | 8.07 |
| *Prompting* | 516 | 26.17 | 541 | 17.56 | 145 | 18.01 |
| *Empathetic utterance* | 65 | 3.3 | 144 | 4.67 | 33 | 4.1 |
| *Alliance bolstering* | 264 | 13.39 | 352 | 11.42 | 197 | 24.47 |
| *Confronting* | 5 | 0.25 | 2 | 0.06 | 0 | 0 |
| *Deadline flexibility* | 6 | 0.3 | 42 | 1.36 | 2 | 0.25 |
| *Emphasizing participant  responsibility* | 1 | 0.05 | 7 | 0.23 | 2 | 0.25 |
| *Self-efficacy shaping* | 3 | 0.15 | 55 | 1.79 | 16 | 1.99 |
| *Informing about modules* | 20 | 1.01 | 61 | 1.98 | 3 | 0.37 |
| *Inadequate detail* | 0 | 0 | 2 | 0.06 | 0 | 0 |
| *Unaddressed content* | 0 | 0 | 1 | 0.03 | 0 | 0 |
| *Unsupportive tone* | 1 | 0.05 | 1 | 0.03 | 0 | 0 |
| *Missed correspondence* | 5 | 0.25 | 17 | 0.55 | 1 | 0.12 |
| *Inappropriate self-disclosure* | 0 | 0 | 0 | 0 | 0 | 0 |
| *Unmanaged risk* | 0 | 0 | 0 | 0 | 0 | 0 |

**Appendix F**

Counts and percentages of therapist behaviors for Completer and Dropout samples.

| **Completers** (number of therapist behaviors, N = 4533) | | | **Dropouts** (number of therapist behaviors, N = 1325) | |
| --- | --- | --- | --- | --- |
|  | n | % | n | % |
| *Clarifying the framework and administrative aspects* | 1083 | 23.89 | 416 | 31.40 |
| *Providing feedback and guidance* | 685 | 15.11 | 110 | 8.30 |
| *Self-disclosure* | 13 | 0.29 | 4 | 0.30 |
| *Psychoeducation* | 156 | 3.44 | 43 | 3.25 |
| *Questions* | 208 | 4.59 | 63 | 4.75 |
| *Reinforcing* | 488 | 10.77 | 79 | 5.96 |
| *Prompting* | 862 | 19.02 | 340 | 25.66 |
| *Empathetic utterance* | 207 | 4.57 | 35 | 2.64 |
| *Alliance bolstering* | 621 | 13.7 | 192 | 14.49 |
| *Confronting* | 5 | 0.11 | 2 | 0.15 |
| *Deadline flexibility* | 39 | 0.86 | 11 | 0.83 |
| *Emphasizing participant  responsibility* | 8 | 0.18 | 2 | 0.15 |
| *Self-efficacy shaping* | 68 | 1.5 | 6 | 0.45 |
| *Informing about modules* | 69 | 1.52 | 15 | 1.13 |
| *Inadequate detail* | 1 | 0.02 | 1 | 0.08 |
| *Unaddressed content* | 1 | 0.02 | 0 | 0 |
| *Unsupportive tone* | 2 | 0.04 | 0 | 0 |
| *Missed correspondence* | 17 | 0.38 | 6 | 0.45 |

**References Supplementary Material**

Hadjistavropoulos, H. D., Gullickson, K. M., Schneider, L. H., Dear, B. F., & Titov, N. (2019). Development of the internet-delivered cognitive behaviour therapy undesirable therapist behaviours scale (ICBT-UTBS). *Internet Interventions, 18*, 100255. https://doi.org/10.1016/j.invent.2019.100255

Holländare, F., Gustafsson, S. A., Berglind, M., Grape, F., Carlbring, P., Andersson, G., ... & Tillfors, M. (2016). Therapist behaviours in internet-based cognitive behaviour therapy (ICBT) for depressive symptoms. *Internet Interventions, 3*, 1–7. <https://doi.org/10.1016/j.invent.2015.11.002>

Schneider, L. H., Hadjistavropoulos, H. D., & Faller, Y. N. (2016). Internet-delivered cognitive behaviour therapy for depressive symptoms: An exploratory examination of therapist behaviours and their relationship to outcome and therapeutic alliance. *Behavioural and Cognitive Psychotherapy, 44*(6), 625–639. <https://doi.org/10.1017/S1352465816000254>
